# Endothelial AMP-Activated Kinase α1 Phosphorylates eNOS on Thr495 and Decreases Endothelial NO Formation

**DOI:** 10.3390/ijms19092753

**Published:** 2018-09-13

**Authors:** Nina Zippel, Annemarieke E. Loot, Heike Stingl, Voahanginirina Randriamboavonjy, Ingrid Fleming, Beate Fisslthaler

**Affiliations:** 1 Institute for Vascular Signalling, Centre for Molecular Medicine, Johann Wolfgang Goethe University, 60590 Frankfurt, Germany; NZippel@gmx.de (N.Z.); a.loot@certe.nl (A.E.L.); Stingl@vrc.uni-frankfurt.de (H.S.); Voahangy@vrc.uni-frankfurt.de (V.R.); fleming@em.uni-frankfurt.de (I.F.); 2 DZHK (German Centre for Cardiovascular Research) partner site RhineMain, Theodor Stern Kai 7, 60590 Frankfurt, Germany

**Keywords:** endothelial nitric-oxide synthase, vasodilation, phenylephrine, vasoconstriction, endothelial cells, ionomycin

## Abstract

AMP-activated protein kinase (AMPK) is frequently reported to phosphorylate Ser1177 of the endothelial nitric-oxide synthase (eNOS), and therefore, is linked with a relaxing effect. However, previous studies failed to consistently demonstrate a major role for AMPK on eNOS-dependent relaxation. As AMPK also phosphorylates eNOS on the inhibitory Thr495 site, this study aimed to determine the role of AMPKα1 and α2 subunits in the regulation of NO-mediated vascular relaxation. Vascular reactivity to phenylephrine and acetylcholine was assessed in aortic and carotid artery segments from mice with global (AMPKα^−/−^) or endothelial-specific deletion (AMPKα^ΔEC^) of the AMPKα subunits. In control and AMPKα1-depleted human umbilical vein endothelial cells, eNOS phosphorylation on Ser1177 and Thr495 was assessed after AMPK activation with thiopental or ionomycin. Global deletion of the AMPKα1 or α2 subunit in mice did not affect vascular reactivity. The endothelial-specific deletion of the AMPKα1 subunit attenuated phenylephrine-mediated contraction in an eNOS- and endothelium-dependent manner. In in vitro studies, activation of AMPK did not alter the phosphorylation of eNOS on Ser1177, but increased its phosphorylation on Thr495. Depletion of AMPKα1 in cultured human endothelial cells decreased Thr495 phosphorylation without affecting Ser1177 phosphorylation. The results of this study indicate that AMPKα1 targets the inhibitory phosphorylation Thr495 site in the calmodulin-binding domain of eNOS to attenuate basal NO production and phenylephrine-induced vasoconstriction.

## 1. Introduction

AMP-activated protein kinase (AMPK) is activated in response to intracellular energy depletion, e.g., during insulin resistance when cellular glucose uptake is limited—especially in contracting skeletal muscle [1] or in cultured cells in the absence of extracellular glucose or hypoxia [2]. Once activated, AMPK acts to conserve energy by stimulating glucose uptake and mitochondrial biosynthesis, as well as by stimulating autophagy to provide substrates for metabolism. At the same time, AMPK inhibits anabolic pathways, such as cholesterol biosynthesis and fatty-acid synthesis, which are not essential for survival (for a recent review, see Reference [3]). In addition to activation by energy-sensitive stimuli, AMPK can also be stimulated following cell exposure to cytokines, growth factors, and mechanical stimuli [4]. In endothelial cells, AMPK was implicated in the inhibition of cell activation [5,6], as well as in angiogenesis in vitro [7] and in vivo [8]. These effects are, at least partially, attributed to the phosphorylation and stimulation of the endothelial nitric-oxide (NO) synthase (eNOS) by AMPK. This claim was backed up by reports of AMPK-dependent phosphorylation of eNOS (on Ser1177) following the exposure of cultured endothelial cells to agonists such as the vascular endothelial growth factor (VEGF) [9] and adiponectin [10], or pharmacological agents including peroxisome proliferator-activated receptor (PPAR) agonists [11] and statins [12]. Similar reports were also published using AMPK activators such as 5-aminoimidazole-4-carboxamide ribonucleotide (AICAR) [13] and metformin [14,15], or natural polyphenols like amurensin G [16] or resveratrol [17]. However, the effects are generally weak and much less impressive than the stimulation seen in response to hypoxia [7], shear stress [18,19,20], and thrombin [21] which result in robust AMPK activation. 

Evidence for a link between AMPK- and NO-dependent alterations in vascular reactivity is also not consistent and depends on the model studied. For example, in resistance arteries in rat hindlimb and cremaster muscles, AICAR induces an NO- and endothelium-independent relaxation [22]. In mice, small-molecule AMPK activators, PT-1 or A769662, elicit the vasodilation of mesenteric arteries by decreasing intracellular Ca^2+^ levels and inducing depolymerization of the actin cytoskeleton [23,24]. In other studies, AICAR was reported to impair the relaxation elicited by sodium nitroprusside (SNP), indicating a general effect on smooth-muscle contractility [25]. In genetic models, the situation is not any clearer as the deletion of the AMPKα1 subunit did not affect acetylcholine (ACh)-induced NO production and relaxation unless mice were treated with angiotensin II over seven days [26]. Also, in isolated phenylephrine-contracted rings of murine aorta, AICAR elicited a profound dose-dependent relaxation that was independent of either the endothelium or the inhibition of eNOS, and mediated by the AMPKα1 subunit in smooth-muscle cells [27]. The most thorough study investigating the role of endothelial AMPKα subunits on vascular function and blood pressure reported hypertension in endothelial-specific AMPKα1 knockout mice; however, in the mesentery artery, the effect was attributed to the opening of charybdotoxin-sensitive potassium channels and smooth-muscle hyperpolarization [28]. The global deletion of the AMPKα2 subunit was also reported to attenuate the ACh-induced relaxation of murine aorta. This effect was attributed to eNOS uncoupling via an AMPKα2-mediated proteasomal degradation of the GTP cyclohydrolase [29], which generates the eNOS cofactor, tetrahydrobiopterin. Also, other researchers failed to detect any evidence for the AMPK-dependent activation of eNOS [30,31]. In this study, we set out to make a more thorough analysis of the effects of AMPKα1 and AMPKα2 deletion on NO-mediated vascular function. We also carefully studied changes in eNOS phosphorylation in cultured and native endothelial cells. 

## 2. Results

### 2.1. Consequences of Global AMPKα Deletion on Vascular Responsiveness

**Figure 1:**
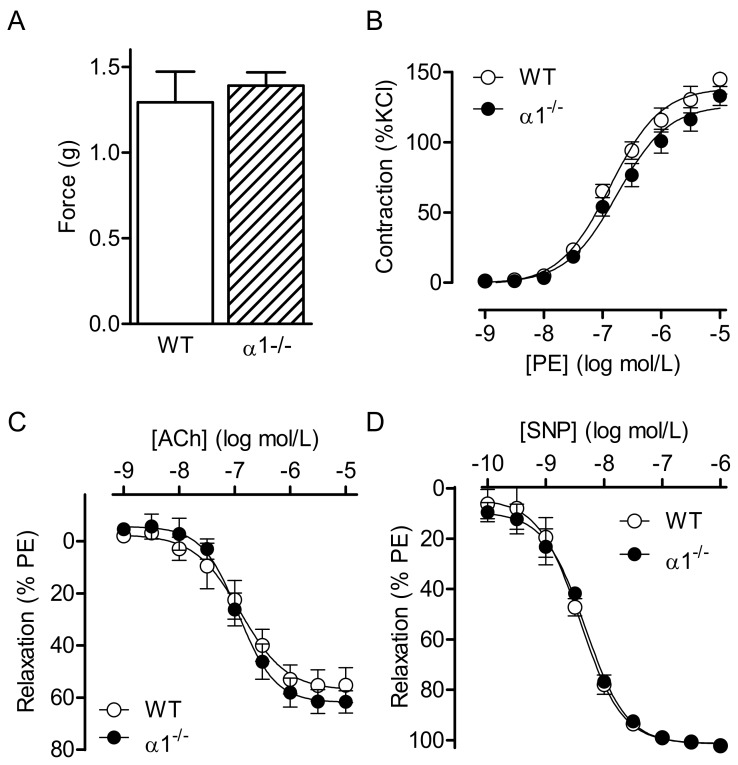
Consequences of global AMP-activated protein kinase (AMPK) α1 deletion on vascular reactivity. Vascular reactivity in aortic rings from wild-type (WT) and AMPKα1^−/−^ (α1^−/−^) mice. (**A**) Responsiveness of endothelium-intact aortic rings to KCl (80 mmol/L). (**B**–**D**) Concentration–response curves to (**B**) phenylephrine (PE), (**C**) acetylcholine (ACh), and (**D**) sodium nitroprusside (SNP). The graphs summarize data obtained from seven animals in each group.

The maximal KCl- and phenylephrine-induced contractions of isolated aortic rings were indistinguishable between wild-type mice and their corresponding AMPKα1^−/−^ littermates (Figure 1A,B). However, there was a tendency toward an attenuated contraction in the aortic rings from the AMPKα1^−/−^ mice and −log half maximal effective concentration (pEC_50_) values were −6.899 ± 0.082 and −6.711 ± 0.099 (*n* = 7, not significant (n.s.)) in rings from wild-type and AMPKα1^−/−^ mice, respectively. The endothelium- and NO-dependent relaxation elicited by ACh (Figure 1C), as well as the endothelium-independent relaxation elicited by SNP (Figure 1D), was identical in vessels from both strains. When experiments were repeated using carotid arteries, samples from AMPKα1^−/−^ mice demonstrated a slightly weaker contractile response to KCl than the wild-type mice, as well as a slightly attenuated response to phenylephrine (pEC_50_ values were −6.393 ± 0.065 and −5.895 ± 0.093, respectively, (*n* = 7, n.s.) in rings from wild-type and AMPKα1^−/−^ mice (Figure S1). Again, there was no apparent difference in relaxant responsiveness to ACh or SNP. 

**Figure 2:**
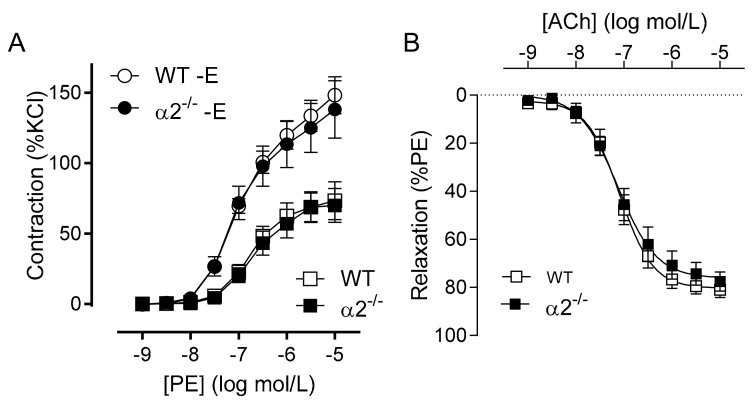
Consequences of global AMPKα2 deletion on vascular reactivity. Vascular reactivity in aortic rings from wild-type (WT) and AMPKα2^−/−^ (α2^−/−^) mice: (**A**) contractile response to phenylephrine (PE) in the presence and absence (−E) of endothelium. (**B**) Concentration-dependent relaxation due to acetylcholine. The graphs summarize data obtained from seven animals in each group.

Similar experiments using arteries from AMPKα2^−/−^ mice gave essentially the same results, i.e., no significant difference in either the agonist-induced contraction or relaxation of the aorta in either the presence or absence of a functional endothelium (Figure 2).

### 2.2. Consequence of Endothelial-Specific AMPKα Deletion on Vascular Responsiveness

As the global deletion of AMPK seemed to affect vascular smooth-muscle contraction rather than NO-mediated relaxation, animals lacking the AMPKα1 or AMPKα2 subunits specifically in endothelial cells (i.e., AMPKα1^ΔEC^ and AMPKα2^ΔEC^ mice) were generated, and the specificity of the deletion verified in isolated cluster of differentiation 144 (CD144)-positive pulmonary endothelial cells (Figure S2A). The deletion of endothelial AMPKα1 did not influence the expression level of AMPKα1 in whole aortic lysates (Figure S2B). 

**Figure 3:**
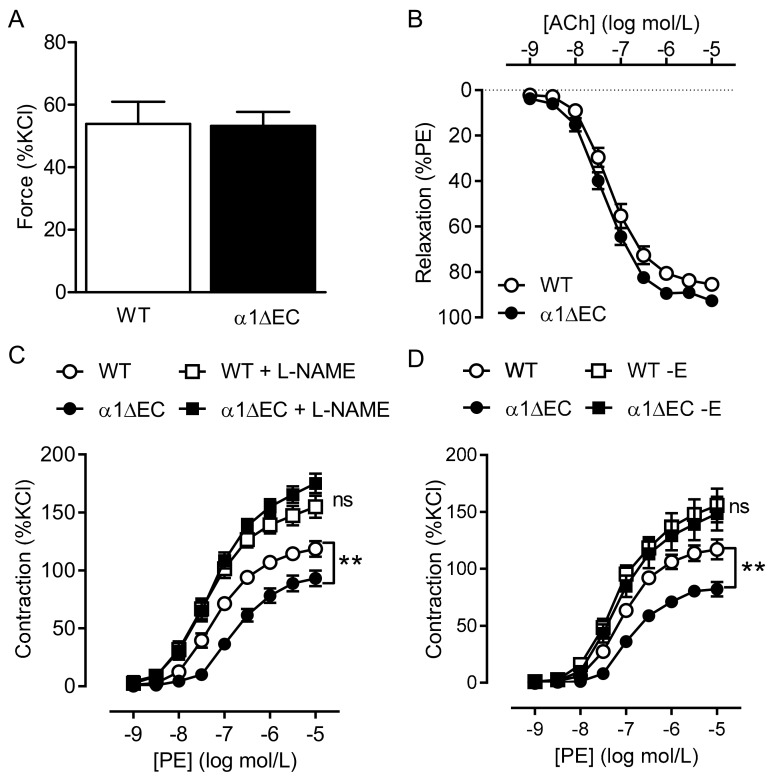
Consequences of endothelial-specific deletion of AMPKα1 on vascular reactivity. (**A**) Effect of N^ω^-nitro-l-arginine methyl ester (L-NAME; 300 µmol/L) on the tone of aortic rings from wild-type (WT) and AMPKα1^ΔEC^ (α1ΔEC) mice pre-contracted to 30% of the maximal KCl-induced contraction by phenylephrine. (**B**) Concentration-dependent relaxation due to acetylcholine in aortic rings pre-constricted with phenylephrine from wild-type (WT) and AMPKα1^ΔEC^ (α1ΔEC) mice. (**C**,**D**) Concentration-dependent contraction of aortic rings from wild-type (WT) and AMPKα1^ΔEC^ (α1ΔEC) mice due to phenylephrine. Experiments were performed in the absence and presence of L-NAME (300 µmol/L, (**C**) and in the presence and absence (−E) of functional endothelium (**D**); *n* = 10 to 16, ** *p* < 0.01 AMPKα1^ΔEC^ versus wild type.

Endothelial-specific deletion of AMPKα1 did not affect the N^ω^-nitro-l-arginine methyl ester (L-NAME)-induced contraction of aortic rings (Figure 3A), which is an index of basal Ca^2+^-independent NO production under isometric stretch conditions [32], or that induced by KCl (not shown). However, the ACh-induced relaxation was slightly improved by endothelial-specific AMPKα1 deletion (Figure 3B) with pEC_50_ values for ACh of −7.217 ± 0.095 and −7.360 ± 0.076 (*n* = 14; n.s.) in rings from wild-type and AMPKα1^−/−^ mice, respectively. An increased production of NO was evident as a markedly impaired contraction of aortic rings from AMPKα1^ΔEC^ mice compared to rings from wild-type mice to phenylephrine that was abolished by L-NAME (Figure 3C, Table 1). Similarly, removal of the endothelium with 3-[(3-cholamidopropyl)dimethylammonio]-1-propanesulfonate (CHAPS) also abrogated the improved relaxation that was dependent on AMPKα1 deletion (Figure 3D, Table 1). 

Endothelial-specific deletion of the AMPKα2 subunit had no consequence on the phenylephrine-induced contraction, ACh-induced relaxation, or the SNP-induced relaxation of isolated aortic rings from wild type versus the respective AMPKα2^ΔEC^ littermates (Figure S3).

### 2.3. Vascular Responses to AMPK Activators

One reason for the lack of consequence of AMPKα1 deletion on agonist-induced relaxation may be related to the fact that the ACh-induced phosphorylation and activation of eNOS is, like that of other agonists, largely regulated by the activity of Ca^2+^/calmodulin-dependent kinase II [33]. Therefore, responses to two potential AMPK activators, i.e., resveratrol [34] and amurensin G [16], as well as two reportedly specific small-molecule AMPK activators, 991 and PT-1, were studied.

Resveratrol elicited the almost complete relaxation of aortic rings from wild-type and AMPKα1^ΔEC^ mice (Figure S4A), but these responses were insensitive to NOS inhibition, and therefore, unrelated to its activation. Amurensin G is reported to activate AMPK in endothelial cells and increase eNOS phosphorylation [16]. While it was able to elicit the NOS inhibitor-sensitive relaxation of aortic rings from wild-type mice, it was equally effective and equally sensitive to NOS inhibition in aortic rings from corresponding AMPKα1^ΔEC^ mice (Figure S4B). Thus, amurensin G exerted its relaxation in an eNOS-dependent manner and the activity of both AMPK activators was AMPKα1-independent. The situation was similar when PT-1 and 991 were studied. The compounds elicited phosphorylation of AMPK in endothelial cells of murine aortic rings from wild-type cells (Figure S4E). Although these compounds elicited vascular relaxation, the responses were slow, and although they were sensitive to NOS inhibition, the effects were comparable in aortic rings from wild-type and AMPKα1^ΔEC^ mice (Figure S4C,D).

### 2.4. AMPKα1 and eNOS Phosphorylation

**Figure 4:**
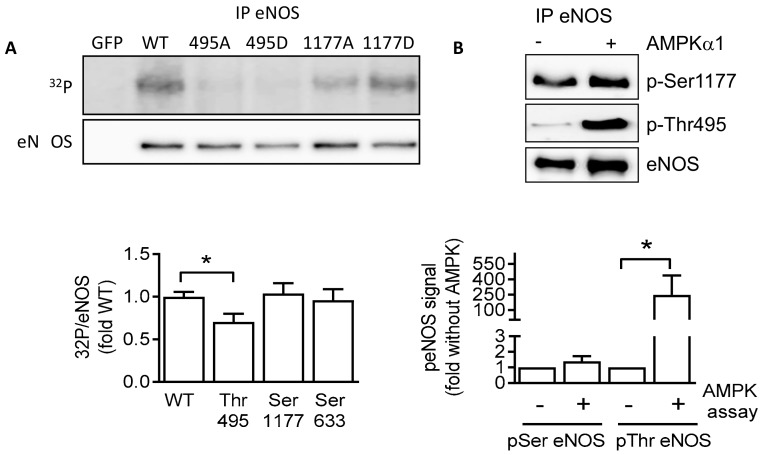
Endothelial nitric-oxide synthase (eNOS) is a substrate of AMPK in vitro. (**A**) Wild-type eNOS, as well as Thr495 and Ser1177 mutants, was overexpressed in HEK293 cells, then immunoprecipitated and used as substrate for AMPKα1 in in vitro kinase assays. The upper panel shows the autoradiograph of eNOS proteins. The lower panel shows the Western blot of the immunoprecipitated (IP) eNOS protein used as input. The graph summarizes the data from four independent experiments. (**B**) Wild-type eNOS (Flag-tagged) overexpressed in human umbilical vein endothelial cells was immunoprecipitated and used as a substrate for AMPKα1. Phosphorylation was assessed using specific antibodies for phosphorylated Ser1177 (p-Ser1177) and Thr945 eNOS (p-Thr495). The graph summarizes the data from five independent kinase reactions. * *p* < 0.05.

The activity of eNOS is reciprocally regulated by its phosphorylation on the activator site, Ser1177 [35,36], and the inhibitory site, Thr495 [37,38,39]. The next step was, therefore, to analyze the ability of AMPK to phosphorylate eNOS on these two residues in vitro. Wild-type (Myc-tagged) eNOS or eNOS mutants in which Thr495 was substituted with alanine or aspartate (Thr495A, Thr495D), or Ser1177 was substituted with alanine or aspartate (Ser1177A, Ser1177D) were overexpressed in HEK293 cells and used as the substrate for in vitro kinase assays for recombinant AMPKα1. While the phosphorylation of wild-type eNOS and Ser1177 mutants was clearly detectable, there was only minimal phosphorylation of the Thr495 mutants (Figure 4A). These findings could be confirmed using phospho-specific antibodies to assess eNOS phosphorylation on Ser1177 and Thr495 on immunoprecipitated eNOS from human endothelial cells (Figure 4B). 

**Figure 5:**
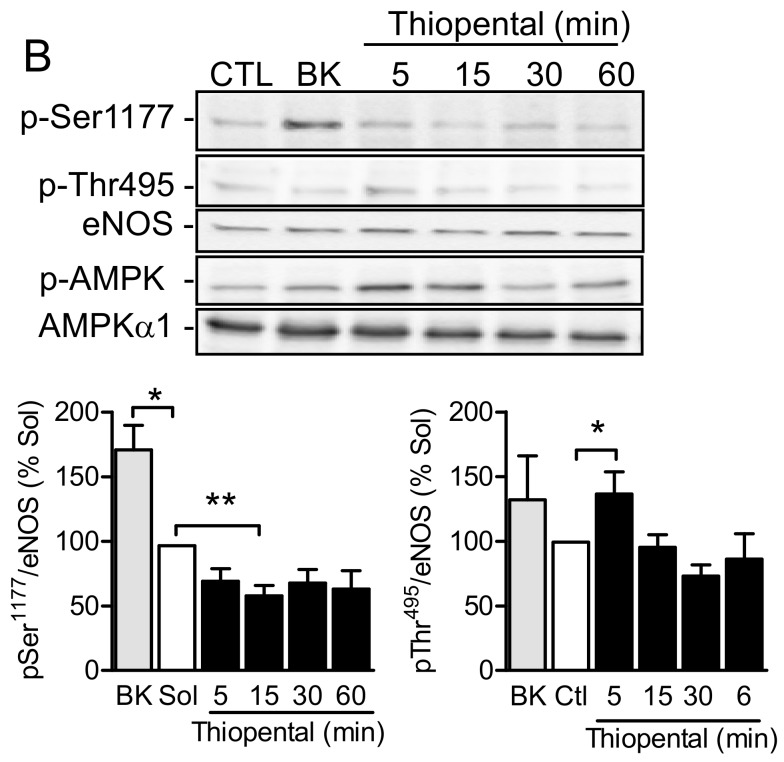
Effect of AMPK activation on eNOS activity and phosphorylation. Cultured human endothelial cells were incubated with solvent (Sol), thiopental (1 mmol/L, 5–60 min), or bradykinin (BK; 1 µmol/L, 2 min). Thereafter, the phosphorylation of (**A**) AMPK and (**B**) phosphorylation of eNOS at Ser1177 and Thr495 were assessed. Bar graphs summarize the data obtained in four to five different cell batches; * *p* < 0.05, ** *p* < 0.01 versus solvent treatment.

To transfer the observations to a more physiological system, changes in eNOS phosphorylation were studied in response to thiopental in cultured endothelial cells. Thiopental elicited the rapid and pronounced phosphorylation of AMPK (Figure 5A) in primary cultures of human endothelial cells. At the same time, thiopental decreased the phosphorylation of eNOS on Ser1177 and increased eNOS phosphorylation on Thr495 (Figure 5B). In contrast, the Ca^2+^-elevating agonist and NO-dependent vasodilator, bradykinin, elicited a significant increase in the phosphorylation of Ser1177, and had no significant effect on Thr495 phosphorylation. 

**Figure 6:**
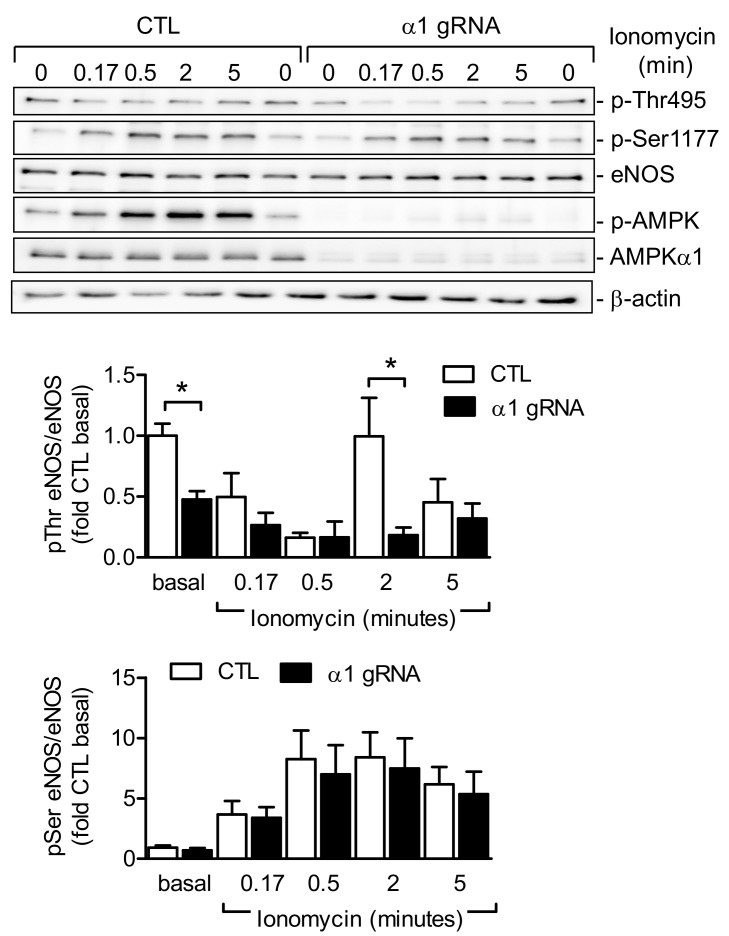
Effect of AMPKα1 deletion on the ionomycin-induced phosphorylation of eNOS on Thr495 and Ser1177. The AMPKα1 subunit was deleted in cultured human cells using the “clustered regularly interspaced short palindromic repeats” (CRISPR)/CRISPR-associated protein 9 (Cas9) system and AMPKα1-specific guide RNAs (gRNAs), and was stimulated with ionomycin (100 nmol/L) for up to 5 min. Representative Western blots are shown of six independent experiments. Bar graphs summarize the evaluation of p-eNOS to total eNOS (*n* = 6); * *p* < 0.05 versus control guide RNA.

Next, AMPKα1 was deleted in human endothelial cells using a “clustered regularly interspaced short palindromic repeats” (CRISPR)/CRISPR-associated protein 9 (Cas9)-based approach. After each passaging, protein expression levels of AMPK α1 were analyzed with Western blotting. After passages 4–5, the protein was no longer detectable (*n* = 6, Figure 6), and agonist-induced changes in eNOS phosphorylation were assessed. Given that bradykinin and ACh receptors are rapidly lost during culture, cells were stimulated with the Ca^2+^ ionophore, ionomycin. In AMPKα1-expressing cells, ionomycin elicited the phosphorylation of eNOS on Ser1177 and the dephosphorylation of Thr495, followed by a rapid re-phosphorylation on Thr495, similar to the effects of other Ca^2+^-elevating agonists [39]. In AMPKα1-depleted cells, basal Thr495 phosphorylation of eNOS was significantly impaired, and the re-phosphorylation after 2 min was also less pronounced than in control cells. The ionomycin-induced phosphorylation of eNOS Ser1177 was not affected by the depletion of AMPKα1 (Figure 6). 

## 3. Discussion

The results of the present study revealed that global deletion of the AMPKα1 or AMPKα2 subunits in healthy animals had no major impact on the relaxant function of isolated endothelium-intact murine aortae or carotid arteries. A small decrease in the contractile response to phenylephrine was apparent in global AMPKα1-deficient arteries, which was much more pronounced following endothelial-specific deletion of the AMPKα1 subunit. Moreover, the attenuated contractile response observed in arteries from AMPKα1^ΔEC^ mice was sensitive to L-NAME and removal of the endothelium, indicating that an increase in NO production by the endothelium underlies the effects observed. Mechanistically, the activity of AMPKα1 could be linked to the phosphorylation of eNOS on the inhibitory Thr495 site. 

There are numerous reports describing the effects on AMPK activators on the phosphorylation of eNOS on Ser1177, which suggests that AMPK acts as an eNOS activator [13,40]. However, most studies linking AMPK with eNOS Ser1177 relied on compound C to inhibit AMPK, or AICAR, phenformin, or resveratrol to activate AMPK. This is a concern as the specificity of these pharmacological tools is questionable, with AMPK-dependent and -independent effects being attributed to both activators and inhibitors [41]. In the present study, the previously reported AMPK activators, amurensin G [16] and resveratrol [34], were studied together with the reportedly more specific small-molecule activators of AMPK, PT-1 [42] and 991 [43], in vascular reactivity studies. No endothelial-specific and AMPKα1-dependent effects were detected using any of the substances tested. Most studies using genetically modified models that reported effects on vascular reactivity focused on global AMPKα-deficient mice, and the defects were usually attributed to vascular smooth-muscle cells [44,45]. Effects on vascular function in global AMPKα1^−/−^ mice were only observed in exercising or angiotensin-II-treated mice. The protective effects of voluntary exercise on vascular function were attributed to AMPKα1 via an effect on eNOS [26,46]. However, the only study to investigate changes in vascular reactivity in vessels from mice lacking AMPKα subunits specifically in endothelial cells linked changes in blood pressure with a carybdotoxin-sensitive potassium channel and endothelial-cell hyperpolarization [28]. 

The majority of reports describing AMPK-mediated effects on vascular function in disease models, as well as in healthy mice, focused on the AMPKα2 subunit, which suppresses reduced nicotinamide adenine dinucleotide phosphate (NADPH) oxidase activity and the production of reactive oxygen species to inhibit 26S proteasomal activity [47]. One consequence of this was the stabilization of GTP cyclohydrolase, the key sepiapterin biosynthetic enzyme that generates the essential eNOS cofactor, tetrahydrobiopterin [29]. It was, therefore, somewhat surprising that no major alterations in NO-mediated relaxation due to ACh could be detected in arteries from animals constitutively lacking the AMPKα2 subunit. Moreover, endothelial-specific deletion of AMPKα2 also failed to affect vascular NO production. 

The enzyme eNOS can be phosphorylated on serine, threonine, and tyrosine residues, findings which highlight the potential role of phosphorylation in regulating eNOS activity. There are numerous putative phosphorylation sites, but most is known about the functional consequences of phosphorylation of a serine residue (human eNOS sequence: Ser1177) in the reductase domain and a threonine residue (human eNOS sequence Thr495) within the calmodulin (CaM)-binding domain. Maximal eNOS activity is linked with the simultaneous dephosphorylation of Thr495 and phosphorylation of Ser1177 [39,48]. 

In unstimulated cultured endothelial cells, Ser1177 is not phosphorylated, but it is rapidly phosphorylated after the application of fluid shear stress [35], VEGF [49] or bradykinin [39]. The kinases involved in this process vary with the stimuli applied. For example, while shear stress elicits the phosphorylation of Ser1177 by protein kinase A (PKA), insulin, estrogen, and VEGF mainly phosphorylate eNOS in endothelial cells via protein kinase B (Akt) [50]. The bradykinin-, Ca^2+^ ionophore-, and thapsigargin-induced phosphorylation of Ser1177 is mediated by Ca^2+^/calmodulin-dependent kinase II (CaMKII) [39]. Thr495, on the other hand, is constitutively phosphorylated in all endothelial cells investigated to date, and it is a negative regulatory site, i.e., phosphorylation is associated with a decrease in enzyme activity [38,39]. The constitutively active kinase that phosphorylates eNOS Thr495 is most probably protein kinase C (PKC) [38], even though there is some confusion regarding the specific isoform(s) involved. AMPK can, however, also phosphorylate Thr495 [37]. The results of this study clearly indicate a role for endothelial cell AMPKα1 in the negative regulation of NO production and vascular tone, and as such, are in line with a previous study that reported an increased NO component to total relaxation in the mesenteric arteries of AMPKα1^ΔEC^ mice compared to wild type [28], this correlated to an enhanced eNOS Thr495 phosphorylation in mesenteric arteries compared to the aorta in wild type mice [51]. Our study also goes further to demonstrate that, in in vitro kinase assays, AMPKα1 clearly phosphorylated eNOS on Thr495, an effect that was prevented by the mutation of Thr495 to Ala or Asp. Also, in AMPKα1-depleted human endothelial cells, basal eNOS phosphorylation on Thr495 was decreased and its re-phosphorylation in response to agonist stimulation was significantly delayed, an effect that can account for the increase in NO generation by AMPKα1-deficient endothelial cells. At this stage, it is not possible to rule out a role for AMPK in the regulation of Ser1177 phosphorylation, as the higher basal phosphorylation of this residue in the transduced cells studied may have masked AMPK-dependent effects. However, the functional studies using vessels from AMPKα1 knockout mice clearly hint at an inhibitory rather than a stimulatory effect of AMPK on eNOS activity. The link between eNOS Thr495 phosphorylation and NO production can be explained by interference with the binding of CaM to the CaM-binding domain. Indeed, in endothelial cells stimulated with agonists such as bradykinin, histamine, or a Ca^2+^ ionophore, substantially more CaM binds to eNOS when Thr495 is dephosphorylated [39]. Analysis of the crystal structure of the eNOS CaM-binding domain with CaM indicates that the phosphorylation of eNOS Thr495 not only causes electrostatic repulsion of nearby glutamate residues within CaM, but may also affect eNOS Glu498, and thus, induce a conformational change within eNOS itself [52]. AMPK activation was also linked with the phosphorylation of eNOS on Ser1177 in isolated endothelial cells [13,37,53], but contrasted somewhat with the lack of effect on endothelium-dependent vascular reactivity [27]. In the present study, only a small increase in Ser1177 phosphorylation was detected in vitro using different cellular sources of eNOS (i.e., HEK cells or human endothelial cells). 

In cultured endothelial cells, we found thiopental to be an effective AMPK activator and could demonstrate that AMPKα1 phosphorylates eNOS on Thr495, an observation that fits well with an earlier report [37]. This phosphorylation step is generally associated with eNOS inhibition due to the decreased binding of Ca^2+^/calmodulin to the enzyme [39], and implies that the activation of AMPK in isolated vessels would act to decrease relaxation and increase vascular tone, which is exactly the response that was observed in the vascular reactivity studies. 

In addition to direct phosphorylation, there are various signaling pathways described for AMPK to influence eNOS activity. AMPK was previously reported to prevent the estradiol-induced phosphorylation of eNOS by preventing the association of eNOS with heat-shock protein 90 (Hsp90), which is generally required for kinase binding to the eNOS signalosome [54]. Any link between AMPK and Hsp90 was not addressed in the current study given the clear effect of AMPKα1 on eNOS phosphorylation in vitro. Direct effects on eNOS activity may not be the only way via which AMPK activation can affect NO signaling. Indeed, AMPKα1 activation could affect the bioavailability of NO by improving mitochondrial function and stimulating the transcriptional regulation of anti-inflammatory enzymes, such as superoxide dismutase 2, to alter the production of reactive oxygen species [55]. 

In summary, endothelial AMPKα subunits have no direct activating effect on eNOS in vivo. Rather, since AMPKα1 phosphorylates eNOS on the inhibitory Thr495 site, AMPK activation attenuates NO production. No link between AMPKα2 and phenylephrine- or ACh-induced changes in vascular tone were detected. Moreover, while some of AMPK activators tested did affect vascular tone, the effects were independent of the endothelial-specific deletion of AMPKα1. 

## 4. Materials and Methods

### 4.1. Materials

The antibodies used were directed against p-Ser1177 (Cell signaling, Cat. No. 9571) and p-Thr495 eNOS (Cell signaling, Cat. No. 9574), eNOS (BD Transduction, 610296), p-Thr172 AMPK (Cell signaling, Cat. No. 2535), AMPKα2 (Cell signaling, Cat. No. 2757), β-actin (Sigma, Cat. No. A5441), Flag (Sigma, Cat. No. F3165), and c-Myc (Santa Cruz, Cat. No. SC-40). The AMPKα1 antibody was generated by Eurogentec by injecting rabbits with the AMPKα1-specific peptide H_2_N–CRA RHT LDE LNPQKS KHQ–CONH_2_. All other substances were obtained from Sigma-Aldrich (Munich, Germany). ^32^Pγ-ATP was obtained from Hartmann Analytics (Braunschweig, Germany). 

### 4.2. Animals

AMPKα1^−/−^ or AMPKα2^−/−^ mice (kindly provided by Benoit Viollet, Paris via the European Mouse Mutant Archive, Munich, Germany) were bred heterozygous and housed at the Goethe University Hospital and knockouts or their respective wild-type littermates were used. AMPKα1^flox/flox^ and α2^flox/flox^ mice with *loxP* sites flanking a coding exon (provided by Benoit Viollet) were crossed with transgenic mouse lines overexpressing Cre recombinase under control of the vascular endothelial (VE)-cadherin promoter to generate the appropriate endothelial-specific AMPKα deletion; Cre^+/−^ mice are referred throughout as AMPKα1^ΔEC^ and AMPKα2^ΔEC^ mice and Cre^−/−^ mice are referred as their respective WT littermates. The investigation conforms to the Guide for the Care and Use of Laboratory Animals published by the European Commission Directive 86/609/EEC. For the isolation of tissues, mice were euthanized with 4% isoflurane in air and subsequent exsanguination.

### 4.3. Vascular Reactivity Measurements

Aortae and carotid arteries were prepared free of adhering tissue and cut into 2.0-mm segments. Aortic rings were mounted in standard 10-mL organ bath chambers, stretched to 1 *g* tension and responses were measured in *g*. Carotid artery rings were mounted in 5-mL wire myograph chambers (DMT, Aarhus, Denmark), stretched to 90% of their diameter at 100 mmHg, and responses were measured in mN/mm segment length. Contractile responses to a high K^+^ buffer (80 mmol/L KCl) or cumulatively increasing concentrations of phenylephrine were assessed. Relaxation to cumulatively increasing concentrations of ACh, resveratrol (Sigma, Munich, Germany), 2-chloro-5-[[5-[[5-(4,5-dimethyl-2-nitrophenyl)-2-furanyl]methylene]-4,5-dihydro-4-oxo-2-thiazolyl]amino]benzoic acid (PT-1; Tocris, Biotechne, Wiesbaden, Germany), amurensin G (kindly provided by K.W. Kang, Seoul, Korea), 5-((6-chloro-5-(1-methyl-1H-indol-5-yl)-1H-benzo [d]imidazol-2-yl)oxy)-2-methylbenzoic acid (991; SpiroChem AG, Switzerland), or SNP was assessed in segments pre-contracted with phenylephrine to 80% of their maximal contraction due to KCl in the presence and absence of L-NAME. Relaxation was expressed as the percentage of phenylephrine precontraction. Removal of the endothelium was performed by intraluminal application of CHAPS (0.5%, 30 s) into the aortae. 

### 4.4. Cell Culture

*Human endothelial cells*: Human umbilical vein endothelial cells were isolated and cultured as previously described [56] and used up to passage 2. The use of human material in this study conforms to the principles outlined in the Declaration of Helsinki, and the isolation of endothelial cells was approved in written form by the ethics committee of Goethe University. For lentiviral and adenoviral transduction, human umbilical vein endothelial cells (Promocell, Heidelberg, Germany) were used and cultured up to passage 8 in endothelial growth medium 2 (Promocell, Heidelberg, Germany).

*Murine pulmonary endothelial cells*: Mouse lungs were freshly processed as previously described [18].

### 4.5. Adenoviral Transduction of Human Umbilical Vein Endothelial Cells

Adenoviral particles expressing the C-terminal Flag-tagged human full-length eNOS were used to transduce cultured umbilical vein endothelial cells as described previously [57].

### 4.6. In Vitro Kinase Assay

The eNOS wild-type or mutant proteins with C-terminal myc or Flag tags were overexpressed by transfection in HEK cells or adenoviral transduction in human umbilical vein endothelial cells, and after two days, cells were lysed and eNOS was immunoprecipitated by c-myc or Flag immunoprecipitation (IP). The immunoprecipitated proteins were used as a substrate for kinase assays with purified AMPKα1/β1/γ1 subunits (Merck Millipore, Darmstadt, Germany, Cat No. 1480) [20]. The lysates were separated by SDS-PAGE and blotted with antibodies specific for the phosphorylation sites of eNOS. Alternatively, ^32^PγATP was used to radioactively label the protein. Proteins were separated by SDS-PAGE, and the gel was exposed to X-ray film after drying. 

### 4.7. CRISPR/Cas9-Mediated Knock-Down of AMPKα1

Human umbilical vein endothelial cells (Promocell, Heidelberg, Germany) were transduced with lentiviral particles mediating the expression of Cas9 (Lenti-Cas9-2A-Blast was provided by Jason Moffat (Addgene plasmid # 73310)) and selected by blasticidin (10 µg/mL). Thereafter, a second lentiviral transduction with guide RNAs directed against AMPKα1 (Addgene numbers 76253 and 75254 provided by David Root, Cambridge, MA, USA) was performed, and puromycin (1 µg/mL) was used to select for double-transduced cells. The efficiency of the knockdown was analyzed by Western blotting. 

### 4.8. Immunoblotting

Cells were lysed in Triton X-100 lysis buffer (Tris/HCl pH 7.5; 50 mmol/L; NaCl, 150 mmol/L; ethyleneglycoltetraacetic acid (EGTA), 2 mmol/L; ethylenediaminetetraacetic acid (EDTA) 2 mmol/L; Triton X-100, 1% (*v*/*v*); NaF, 25 mmol/L; Na_4_P_2_O_7_, 10 mmol/L; 2 μg/mL each of leupeptin, pepstatin A, antipain, aprotinin, chymostatin, and trypsin inhibitor, and phenylmethylsulfonyl fluoride (PMSF), 40 μg/mL). Detergent-soluble proteins were heated with SDS-PAGE sample buffer and separated by SDS-PAGE, and specific proteins were detected by immunoblotting. To assess the phosphorylation of proteins, either equal amounts of protein from each sample were loaded twice and one membrane incubated with the phospho-specific antibody and the other with an antibody recognizing total protein, or blots were reprobed with the appropriate antibody. 

### 4.9. Statistical Analyses

Data are expressed as mean ± standard error of the mean (SEM). Statistical evaluation was done using Student’s *t*-test for unpaired data or ANOVA for repeated measures where appropriate. Values of *p* < 0.05 were considered statistically significant.

## Figures and Tables

**Table 1:** The −log half maximal effective concentration (pEC_50_) values relating to the consequences of endothelial-specific deletion of AMP activated protein kinase (AMPK) α1 on vascular response to phenylephrine. Experiments were performed in endothelium intact rings the presence of solvent or N^ω^-nitro-l-arginine methyl ester (L-NAME; 300 µmol/L), as well as in endothelium-denuded (−E) samples from the same animals; *n* = 10–16.

pEC_50_ Values	Solvent		L-NAME		−E	
Wild type	−7.04 ± 0.13		−7.43 ± 0.10		−7.21 ± 0.05	
AMPK^ΔEC^	−6.77 ± 0.05	*	−7.31 ± 0.09	§§	−7.13 ± 0.05	§§

* *p* < 0.05 versus wild type; §§ *p* < 0.001 versus solvent.

## Supplementary Materials

Supplementary materials can be found at http://www.mdpi.com/1422-0067/19/9/2753/s1. Figure S1. Vascular function in carotid arteries from wild-type (WT) and AMPKα1^−/−^ mice. (A) Contraction induced by KCl (80 mmol/L), (B) concentration response curves to phenylephrine (PE), and relaxation curves to (C) acetylcholine (ACh) or (D) sodium nitroprusside (SNP) in PE-contracted vessels. The graphs summarize data obtained from 7 animals in each group. Figure S2. Endothelial cell specific deletion of AMPKα1. (A) AMPKα1 expression in freshly isolated pulmonary endothelial cells from AMPKα1^ΔEC^ or Cre^−/−^ (wild-type; WT) mice. (B) Expression of eNOS, AMPKα1 and AMPKα2 in aortic ring lysates from WT or AMPKα1^ΔEC^ (ΔEC) mice. (A) The blots presented are representative of 12 additional experiments using 2 mice per group. Figure S3. Effect of endothelial specific deletion of AMPKα2 on vascular reactivity of aortic rings (A) Dose dependent contraction to PE of wild-type (open symbols) or AMPKα2^ΔEC^ mice (closed symbols). (B) Relaxation curves of aortic rings to acetylcholine (ACh) after PE constriction of wild-type (open symbols) or AMPKα2^ΔEC^ mice (closed symbols). (C) Dose-dependent relaxation to SNP. The graphs summarize data obtained from 6 animals in each group. Figure S4. Effect of AMPK activators on the relaxation of aortic rings. (A,B) Concentration dependent effects of resveratrol (A) and amurensin G (B) on vascular tone in phenylephrine preconstricted aortic rings from wild-type (WT) and AMPKα1^ΔEC^ (α1^ΔEC^) mice; *n* = 6 animals in each group. (C,D) Time-dependent effects of PT-1 (30 µmol/L) and 991 (30 µmol/L) on vascular tone in phenylephrine preconstricted aortic rings from wild-type (WT) and AMPKα1^ΔEC^ (α1^ΔEC^) mice; *n* = 4 animals in each group. (E) Effects of the AMPK activators on the phosphorylation of AMPK (on Thr172) and ACC (Ser79) in endothelial cells isolated from aortic rings from wild-type mice. Experiments were performed in the absence (Basal) and presence of 991 (30 µmol/L), AICAR (0.5 mmol/L) or PT-1 (30 µmol/L) for 60 min. Comparable results were obtained in 3 additional independent experiments.

## Abbreviations

AMPK	AMP-activated protein kinase
eNOS	endothelial nitric-oxide synthase
AICAR	5-aminoimidazole-4-carboxamide ribonucleotide
VEGF	vascular endothelial growth factor
PPAR	peroxisome proliferator-activated receptor
SNP	sodium nitroprusside
ACh	acetylcholine
PE	phenylephrine
L-NAME	N^ω^-nitro-l-arginine methyl ester
CRISPR	clustered regularly interspaced short palindromic repeats
Cas9	CRISPR-associated protein 9
