# Supplementary material for: Endothelial AMP-Activated Kinase α1 Phosphorylates eNOS on Thr495 and Decreases Endothelial NO Formation
Source: Int J Mol Sci. 2018 Sep 13;19(9):2753. doi: 10.3390/ijms19092753 (PMC6165563; doi:10.3390/ijms19092753)
Supplement: Supplementary file 1 [file ijms-19-02753-s001.pdf]

Endothelial AMP-Activated Kinase α1 Phosphorylates eNOS on Thr495 and Decreases Endothelial NO Formation

Nina Zippel [1], Annemarieke E. Loot [1], Heike Stingl [1,2], Voahanginirina Randriamboavonjy [1,2], Ingrid Fleming[1,2] and Beate FissIthaler[1,2,*]

[1] Institute for Vascular Signalling, Centre for Molecular Medicine, Johann Wolfgang Goethe University, 60590 Frankfurt, Germany; NZippel@gmx.de (N.Z.); a.loot@certe.nl (A.E.L.); Stingl@vrc.uni-frankfurt.de (H.S.); Voahangy@vrc.uni-frankfurt.de (V.R.); fleming@em.uni-frankfurt.de (I.F.)

[2] DZHK (German Centre for Cardiovascular Research) partner site RhineMain, Theodor Stern Kai 7, 60590 Frankfurt, Germany

* Correspondence: fisslthaler@vrc.uni-frankfurt.de; Tel.: +49-69-6301-6994

Supplementary Materials:

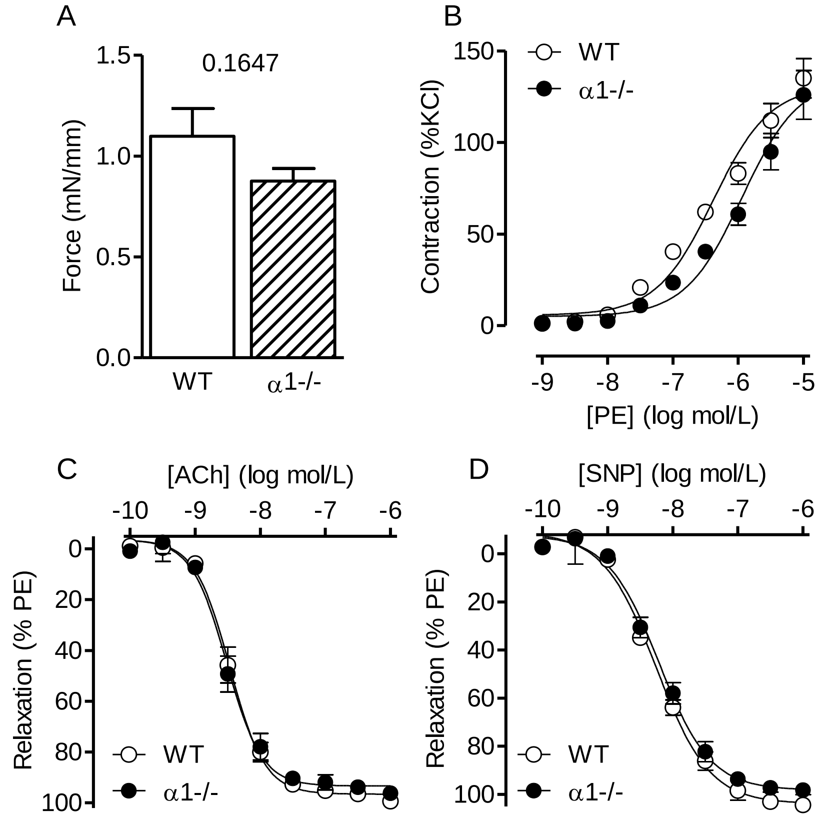

Figure S1. Vascular function in carotid arteries from wild-type (WT) and AMPKα1$^{-/-}$ mice. (**A**) Contraction induced by KCl (80 mmol/L), (**B**) concentration response curves to phenylephrine (PE), and relaxation curves to (**C**) acetylcholine (ACh) or (**D**) sodium nitroprusside (SNP) in PE-contracted vessels. The graphs summarize data obtained from 7 animals in each group.

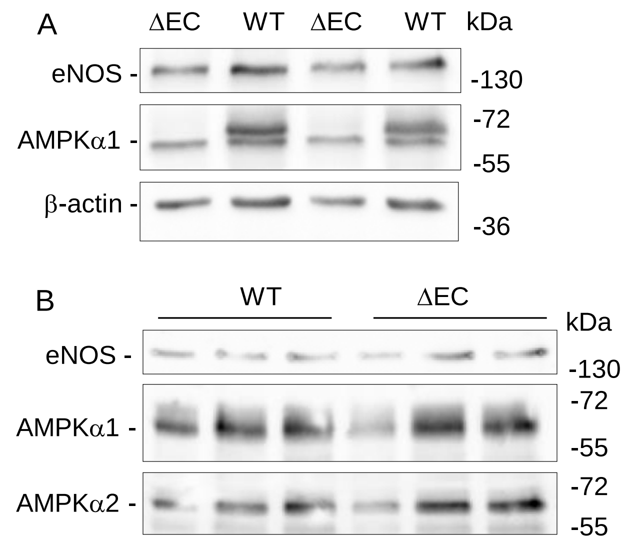

Figure S2. Endothelial cell specific deletion of AMPKα1. (**A**) AMPKα1 expression in freshly isolated pulmonary endothelial cells from AMPK$\alpha 1^{\Delta EC}$ or $Cre^{-/-}$ (wild-type; WT) mice. (**B**) Expression of eNOS, AMPKα1 and AMPKα2 in aortic ring lysates from WT or AMPK$\alpha 1^{\Delta EC}$ (ΔEC) mice. (**A**) The blots presented are representative of 12 additional experiments using 2 mice per group.

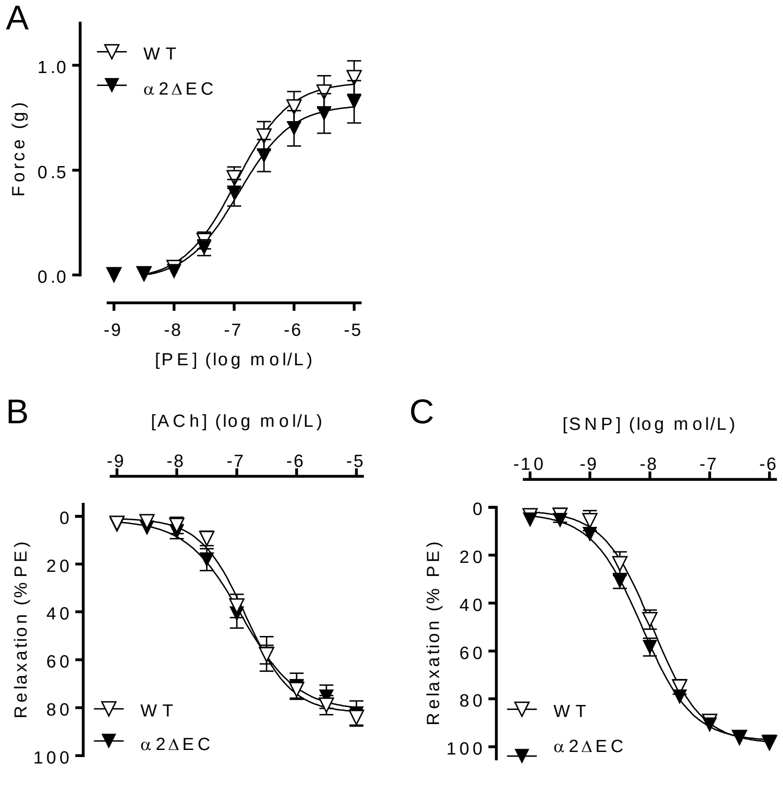

Figure S3. Effect of endothelial specific deletion of AMPKα2 on vascular reactivity of aortic rings (**A**) Dose dependent contraction to PE of wild-type (open symbols) or AMPK$\alpha 2^{\Delta EC}$ mice (closed symbols). (**B**) Relaxation curves of aortic rings to acetylcholine (ACh) after PE constriction of wild-type (open

symbols) or AMPK$\alpha2^{\Delta EC}$ mice (closed symbols). (**C**) Dose-dependent relaxation to SNP. The graphs summarize data obtained from 6 animals in each group.

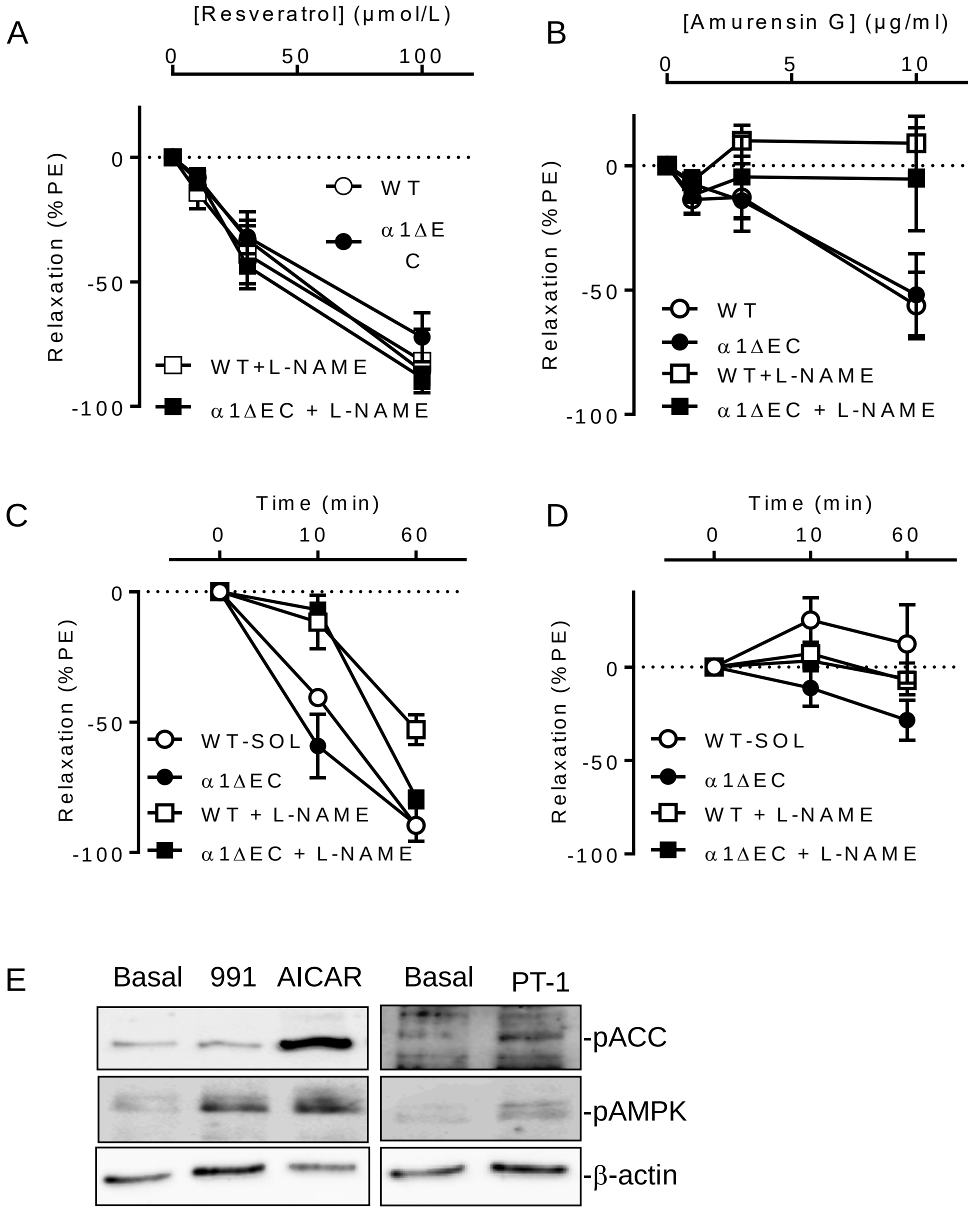

Figure S4. Effect of AMPK activators on the relaxation of aortic rings. (**A,B**) Concentration dependent effects of resveratrol (**A**) and amurensin G (**B**) on vascular tone in phenylephrine preconstricted aortic rings from wild-type (WT) and AMPK$\alpha1^{\Delta EC}$ ($\alpha1^{\Delta EC}$) mice; n = 6 animals in each group. (**C,D**) Time-dependent effects of PT-1 (**C**, 30 μmol/L) and 991 (**D**; 30 μmol/L) on vascular tone in phenylephrine preconstricted aortic rings from wild-type (WT) and AMPK$\alpha1^{\Delta EC}$ ($\alpha1^{\Delta EC}$) mice; n = 4 animals in each group. (**E**) Effects of the AMPK activators on the phosphorylation of AMPK (on Thr172) and ACC (Ser79) in endothelial cells isolated from aortic rings from wild-type mice. Experiments were performed in the absence (Basal) and presence of 991 (30 μmol/L), AICAR (0.5 mmol/L) or PT-1 (30 μmol/L) for 60 min. Comparable results were obtained in 3 additional independent experiments.
